# The effect of prolonged exposure to war-related stress among hospital personnel with different affect types: lessons from the Second Lebanon War and the Gaza “Cast Lead” operation

**DOI:** 10.3402/ejpt.v3i0.7165

**Published:** 2012-10-25

**Authors:** Yuval Palgi, Menachem Ben-Ezra, Amit Shrira

**Affiliations:** 1 Department of Gerontology, Faculty of Social Welfare and Health Sciences, University of Haifa, Haifa, Israel; 2 School of Social Work, Ariel University Center of Samaria, Ariel, Israel; 3 The Interdisciplinary Department of Social Sciences, Bar-Ilan University, Ramat-Gan, Israel

**Keywords:** PTSD, Second Lebanon War, Cast Lead, hospital personnel, negative affect, positive affect

## Abstract

Two studies examined peritraumatic symptoms due to war-related stress among hospital personnel with different affect types. In Study 1, we examined 80 Israeli hospital personnel during the period they were exposed to frequent missile attacks in the Second Lebanon War. In Study 2, we examined 67 and 74 Israeli hospital personnel during the time they were exposed and were not exposed, respectively, to missile attacks in the Gaza “Cast Lead” operation. In both studies, hospital personnel completed measures of posttraumatic stress disorder symptoms as well as of positive- and negative-affect items (PA and NA, respectively). Exposed personnel with a positive congruent (high PA and low NA) or a deflated incongruent (low PA and low NA) affective types had a lower level of peritraumatic symptoms compared to those with a negative congruent (low PA and high NA) or an inflated incongruent (high PA and NA) affective types. Study 2 further showed that among non-exposed personnel, only personnel with a negative congruent affective type had a higher level of peritraumatic symptoms compared to personnel with other affective types. Clinical implications and required future studies are discussed.

## Posttraumatic stress disorder

A substantial amount of literature is available on the harmful effects of exposure to extreme traumatic stress. Although about 60% of the civilian population in the United States is exposed to a traumatic event during their life, the lifetime prevalence of posttraumatic stress disorder (PTSD) was roughly 7% (Brewin, Andrews, & Valentine, 2000; Ozer, Best, Lipsey, & Weiss, 2003). PTSD is related to impairment in major functioning facets (physical, emotional, cognitive, and social) as well as to other psychiatric conditions such as depression, anxiety, and somatic symptoms (Kessler, 2000; Yehuda, 2002). Moreover, according to the dose–response model, repeated and continued extreme exposure to casualties and dead bodies is directly related to a worsened functioning (March, 1993). Nevertheless, some conditions and characteristics were found to help specific individuals to be more resilient in the face of exposure to traumatic experiences (Ben-Ezra & Soffer, 2010; Ben-Ezra et al., 2011; Fredrickson, 2001).

Practitioners in the caring professions show a relative resilience and even posttraumatic growth to extreme stress; yet they too report considerable distress during adversity (Lev-Wiesel, Goldblatt, Eisikovitz, & Admy, 2009). Being under direct threat toward one's self and family members while treating others is known to enhance burnout, emotional distress, and other negative consequences among mental health workers (Dekel & Baum, 2009). Previous studies examined hospital personnel who were indirectly exposed to specific traumatic events by caring for victims of bombings, terror attacks, and sniper shootings (Dekel, Hantman, Ginzburg, & Solomon, 2007; Grieger, Fullerton, Ursano, & Reeves, 2003; Kerasiotis & Motta, 2004; Luce, Firth-Cozens, Midgley, & Burges, 2002; Weiniger et al., 2006), but only recent works assessed the health of personnel who were directly exposed to intense, prolonged war stress (Ben-Ezra, Palgi, & Essar, 2007, 2008; Essar, Ben-Ezra, Langer, & Palgi, 2008; Palgi, Ben-Ezra, Langer, & Essar, 2009). In contrast to previous studies (Bryant & Harvey, 2000; Mayou, Ehlers, & Hobbs, 2000), these recent works examined hospital personnel during (and not after) the stressful event. However, although the previous studies examined mostly traumatic symptoms that predict well-being, the present studies refer to well-being as a mode of adaptation and therefore as an independent variable.

## Subjective well-being

Subjective well-being (SWB) broadly refers to personal evaluations that people make about their lives. It is a multifaceted construct involving affective, cognitive, and temporal dimensions (Diener, 1984; Diener, Suh, Lucas, & Smith, 1999; Larsen & Eid, 2008). Recent findings indicate that different SWB dimensions (e.g., affective and cognitive) or intradimensional components (e.g., positive and negative affect, hereafter referred to as PA and NA, respectively) do not necessarily reflect each other and can fluctuate separately from each other (Diener, Lucas, & Scollon, 2006). Shmotkin (1998, 2005) suggested that by cross-tabulating different SWB dimensions or intradimensional components, one can better capture the differential nature of SWB. Thus, the emotional affective typology has been delineated by cross-tabulating PA and NA as presented ahead. The present studies focuses on this typology.

## Affect typology

**Fig. 1:**
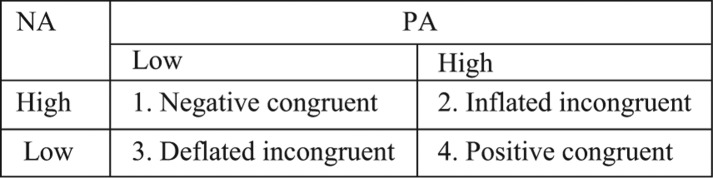
Affect typology according to Shmotkin (1998, 2005).

The cross-tabulation of low and high PA and NA levels creates four affective types: two congruent (cells 1 and 4) and two incongruent types (cells 2 and 3) (see Fig. 1).

The congruent types reflect a dominance of one affect over the other and, therefore, can be characterized by a consistent affective valence. On contrary, the incongruent types do not reflect the dominance of one affect and, therefore, can be characterized by an inconsistent affective valence, whether when both affects are high (cell 2) or low (cell 3).

The positive congruent type is probably the most common type as national surveys demonstrated that “most people are happy” (Diener & Diener, 1996). The common predominance of PA over NA is a part of a basic “positivity bias” that has evolutional advantages in preferring approach over avoidance tendencies in normal, non-stressful, conditions (Cacioppo, Gardner, & Bernston, 1999; Larsen, Hemenover, Norris, & Cacioppo, 2003) as well as in moderately stressful and even traumatic situations (Fredrickson, Tugade, Waugh, & Larkin, 2003; Hassett et al., 2008; Robertson, Zarit, Duncan, Rovine, & Femia, 2007; Tugade & Ferdrickson, 2004).

The negative congruent type has fewer advantages in functional terms, especially when including sadness and depression, which are associated with stressful circumstances, physical illness, and general vulnerability (Hassett et al., 2008; Robertson et al., 2007; Zeidner, 2006). Nevertheless, under certain circumstances, the predominance of NA over PA may have some advantages, especially when NA predominantly includes feelings of fear and anxiety (Baumeister, Bratslavsky, Finkenauer, & Vohs, 2001; Carver, 2004; Parrott, 1993; Rozin & Royzman, 2001).

What is defined here as inflated incongruent type was found to signal uncomfortable and bewildering feelings in normal situations, but was also a useful and efficient affective pattern in coping with high, but not extremely high, stressful situations (Folkman, 1997; Larsen et al., 2003).

The deflated incongruent type reflects “co-inhibition”, which is associated with indifference and apathy (Larsen, McGraw, & Cacioppo, 2001) or with a lack of energy expressed in affective numbness and dissociation following traumatic events (Lifton, 1988). Even so, it can be an adequate mode of adaptation during the process of prolonged stress such as chronic disease (Hassett et al., 2008).

To sum up, affective types may be defined as a personally constructed configuration of SWB with different relations between PA and NA. In the face of stressful situation, these types represent in many ways modes of adaptation that can differently affect the development of PTSD symptoms. The congruent types represent a reciprocal model of affective activation in which PA and NA tend to be mutually exclusive (Cacioppo et al., 1999). When confronted with adversity, individuals with a positive congruent type possibly possess the personal resources that help them to dismantle psychological adversity. The negative congruent type probably indicates a failure in emotional regulation and is expected to be found among those who react in maladaptive ways (Zeidner, 2006). The incongruent types reflect a dialectical mode of adaptation where contradicting affects co-function (Cacioppo et al., 1999; Shmotkin, Berkovich, & Cohen, 2006). The inflated incongruent type signals an effective strategy in handing emotionally complex situations (Folkman & Moskowitz, 2000; Larsen et al., 2003) and, therefore, is expected to be advantageous under adversity. The deflated incongruent type marks a flattened emotional experience, possibly due to a defensive inhibition (e.g., suppression, affective dissociation). A deflated emotional experience is less adaptive in everyday situations, or in the aftermath of a traumatic event, but it might have certain protective properties when confronting traumatic experiences (Campbell, Baumeister, Dhavale, & Tice, 2003; Hassett et al., 2008; Lifton, 1988).

The present studies aim to delineate different affect types, which serve as modes of adaptation under stressful situations. These studies claim that under extreme stressful situations different types result in different levels of traumatic symptoms. Such a finding may have practical implications for hospital-personnel policy under extreme stressful situations.

## Hypotheses

The study's hypotheses refer to the relation of the four affective types to posttraumatic symptoms among hospital personnel working in a long-lasting war stress situation. Exposed personnel with a positive congruent affective type (high PA and low NA) and inflated incongruent type (high PA and high NA) were expected to report a lower level of posttraumatic symptoms than those with a negative congruent type (low PA and high NA), while those with a deflated incongruent type (low PA and low NA) were expected to have an intermediate level of posttraumatic symptoms. Personnel with a negative congruent affective type were expected to report the highest posttraumatic symptoms level. Among unexposed personnel, we expected that those with a positive congruent affective type would report a lower level of posttraumatic symptoms compared to the other three types.

## Method

### Study 1

#### Event

On July 12, 2006, at 9:30 am, war erupted between Israel and Lebanon. Israel suffered 163 fatalities (44 civilians and 119 soldiers) and 2,400 wounded (2,000 civilians and 400 soldiers). During the war, the northern city of Haifa was targeted by hundreds of missiles. The Rambam hospital, located in Haifa, is the largest and most important hospital in northern Israel and serves a population of 1 million people with 75,000 hospitalizations and 500,000 admissions each year. A large proportion of the civilians and military casualties were admitted to that hospital during the war. The hospital itself was also targeted, with 40 missiles landing in the hospital vicinity.

#### Participants

A sample of hospital personnel were selected at random in the fifth week after the war begun. This was done in order to be able to assess PTSD symptoms in the previous month. The initial sample included 109 participants. The response rate was 80%. The most common reason for refusing was “no time”. The final sample (after excluding participants who had partially completed the survey) consisted of 80 participants (their mean age was *M*=35.07 SD=8.81; range 23–60; 30% were men, *n*=24; 65% married *n*=52; and 48% physicians *n*=38). There were no differences between respondents and non-respondents in demographic data (age, gender, and profession). None of the participants reported a history of mental disorders and prior exposure to war-related stress. All the participants were under missile attacks with immediate threat to life and exposure to war casualties, both military and civilian (for more details see Ben-Ezra et al., 2007, 2008; Essar et al., 2008; Palgi et al., 2009).

#### Measures

*Demographic measures*. Age, gender, marital status, profession, and religiosity were assessed with a short demographic questionnaire. For statistical reasons, we dichotomized marital status to married (including married with or without children) and not married (including single, widow, or divorced). Religiosity was dichotomized to secular and religious (including conservative and orthodox). Years of education were not assessed as all participants had academic education, and this variable represented mostly profession differences.

*Positive affect and negative affect*. PA and NA were measured by two subscales from the Center for Epidemiologic Studies-Depression (CES-D) (Radloff, 1977). This questionnaire includes 20 items and is widely used as a major marker of mental health (Stansbury, Ried, & Velozo, 2006). This questionnaire contains four subscales (Gatz & Hurwicz, 1990). In the present study only the “lack of well-being” subscale (containing PA items which are usually reverse coded to denote lack of well-being) and the “depressed mood” subscale were used for PA and NA, respectively. The use of the “depressed mood” subscale for measuring NA is very common. Although less prevalent, there are several studies who used the “lack of well-being” subscale (without reversing it) to measure PA (Diwan, Jonnalagadda, & Balaswamy, 2004; Miller, Markides, & Black, 1997; Ostir, Ottenbacher, & Markides, 2004; Park-Lee, Fredman, Hochberg, & Faulkner, 2009; Schmutte & Ryff, 1997; Sheehan, Fifield, Reisine, & Tennen, 1995).

The PA and NA CES-D subscales are known as good indices of the affective SWB dimension (Schmutte & Ryff, 1997; Sheehan et al., 1995). Seven items were used, as accepted, for the experience of NA (e.g., “I felt depressed”) and four for the PA (e.g., “I felt happy”). The items were rated for their frequency during the previous month on a scale of 0 (*not at all*), 1 (*sometimes*), 2 (*most of the time*), and 3 (*almost every day*) and PA and NA scores were the participant's mean ratings on the two respective subscales. In the present study, Cronbach's alpha for the PA subscale was 0.56. As the item “I felt that I was just as good as other people” considerably lowered the internal reliability, it was decided to omit this item and construct the PA subscale from the other three items. The Cronbach's alpha of the new three-item PA subscale was 0.75. In the present study, the Cronbach's alpha coefficient for the seven-item NA subscale was 0.85. The two subscales did not significantly correlate (*r*=−0.17).

*PTSD symptoms*. Were assessed with the 22-item Impact of Event Scale-Revised (IES-R) (Weiss & Marmar, 1997), which examines the severity of intrusion, avoidance, and hyperarousal symptoms over the past week on a 5-point severity scale: 0 (not at all) to 4 (extremely). In the present study, Cronbach's alpha for the total IES-R was 0.92. The Cronbach's alpha for the IES-R subscales, intrusion, avoidance, and hyperarousal, was 0.89, 0.84, and 0.85, respectively. PTSD symptom scores were the participant's sum ratings on the IES-R. Intrusion, avoidance, and hyperarousal scores were the participant's sum rating on the respective subscales. In several analyses, we used the sum ratings and in others we used a cutoff score to differentiate participants below and above the clinical level of PTSD symptoms. In the latter case, a total score equal or higher than 33 indicated a clinical level of PTSD symptoms (Creamer, Bell, & Failla, 2003).

#### Procedure

The study took the form of a short screening interview and survey. The participants were located and interviewed by the interviewers when they arrived to a safe shelter due to rocket attack alarms as they had no time to fill questionnaires during their shifts. The participants were asked about their profession and age, and asked for their consent to answer the questionnaire. The interview lasted about 15 min and the interviewers helped the participants with unclear items. This study was formally approved according to the Helsinki Committee's ethical requirements (IRB) in Tel Aviv University and was part of a survey regarding hospital personnel under stressful situations.

### Study 2

#### Event

On December 27, 2008, Operation “Cast Lead” (also known as the Gaza War) erupted. During the military engagements between Israel and Hamas, more than 750 rockets were shot into southern Israel, about 100 of which targeted the city of Ashkelon. This operation erupted after 8 years of persistent rockets. The unsheltered Barzilai Medical Center in Ashkelon was under direct rocket attacks while treating 616 people (193 Israeli soldiers, and 423 Israeli and Palestinian civilians) admitted to the hospital as a result of the armed conflict. This situation led to extreme pressure on the hospital personnel, already under immediate risk to their own lives as well as to their families who lived in the greater Ashkelon area. At the same time, being far from the missile range of the Hamas forces, hospital personnel from the Sourasky Medical center at Tel-Aviv were not involved in any war stress situation and routinely treated patients that were admitted to the hospital.

#### Participants

Two groups of hospital personnel were selected at random in both hospitals during the days of January 12–15, 2009 (3 weeks after the operation begun). Later on, the two groups were matched for age, gender, marital status, profession, and religion. Two physicians were excluded from the exposed group as they were part of the board of governors and performed only managerial and administrative work. The response rate was 85% at Barzilai Medical Center and 90% at Sourasky Medical Center. Those who declined were asked about their reasons for refusal. The most frequent reason given for not participating was related to shortage of time. The final group from Barzilai Medical Center (hereafter referred to as “exposed”) included 67 participants (mean age 40.67, SD=10.11; range 25–60; 50 women; 47 married; 21 physicians). The final group from Sourasky Medical Center (hereafter referred to as “unexposed”) included 74 participants (mean age 38.46, SD=9.45; range 24–60; 51 women; 46 married; 33 physicians). The two groups did not differ in background demographics except education, favoring unexposed medical personnel (*t*=−2.365; *p*=0.019). The study took the form of a short screening interview and survey. All the participants were guaranteed complete anonymity and were interviewed incognito.

#### Measures

The measures were identical to those used in Study 1. The Cronbach's alpha of the three-item PA subscale was 0.73 and 0.76 for the exposed and unexposed group respectively. The Cronbach's alpha coefficients for the seven-item NA subscale were 0.80 and 0.81 for the exposed and unexposed, respectively. There was a small non-significant correlation between the two subscales (*r*=−0.11 and −0.22 for the exposed and unexposed group, respectively). The Cronbach's alpha for the IES-R was 0.88 and 0.93 for the exposed and unexposed group respectively. In the exposed group the Cronbach's alpha for the IES-R subscales, intrusion, avoidance, and hyperarousal was 0.73, 0.85, and 0.73, respectively. In the unexposed group, the Cronbach's alpha for the intrusion, avoidance, and hyperarousal subscales was 0.85, 0.87 and 0.79, respectively.

#### Procedure

The participants were interviewed in their wards during their shifts. The participants were asked about their profession and age, and asked for their consent to answer the questionnaire. The interviews lasted about 15 min and the interviewers helped the participants with unclear items. The inclusion criteria were:

1. No history of severe health problems, mental disorders (including stress-related disorders or prior depression), substance abuse, and no prior exposure to severe emotional trauma or to war-related trauma.
2. Treating patients during the period of the Cast Lead operation.
3. Both the hospital personnel and her/his kin family reside in the same area. This study was formally approved according to the Helsinki Committee's ethical requirements (IRB) in Tel Aviv University.

#### Data analysis

Affective types were classified according to combinations of high and low PA and NA (high and low levels were determined using the median split of the PA and NA distribution in every sample, as done by Robertson et al., 2007). Affective type membership was determined by crossing the low and high dimensions of PA and NA. This resulted in four affective types, as presented before (Robertson et al., 2007; Shmotkin, 2005).

Univariate and multivariate analyses of covariance (ANCOVA and MANCOVA) were used to compare the affective types in total score of PTSD symptoms and in intrusion, avoidance, and hyperarousal scores. Age, gender, marital status, religiosity, and profession (physicians and nurses) were held as covariates. Subsequent Chi-square analyses were used to assess the distribution of participants with a clinical level of PTSD symptoms among the affective types. All analyses were conducted using the SPSS program (SPSS, version 15, Chicago, IL).

## Results

Table 1 presents the distribution of the demographics among the affective types in both studies.

**Table 1:** Demographic characteristics of the affective types

	Negative congruent	Positive congruent	Deflated incongruent	Inflated incongruent		
Study 1 (Second Lebanon War)	(*n*=27)	(*n*=18)	(*n*=17)	(*n*=15)		
Study 2 (Gaza War Exposed)	(*n*=19)	(*n*=16)	(*n*=15)	(*n*=17)		
(Gaza War Unexposed)	(*n*=26)	(*n*=13)	(*n*=15)	(*n*=20)	Test statistics	*p*
Age, y (SD)						
Study 1 (Second Lebanon War)	35.98 (8.1)	35.89 (9.2)	34.47 (9.9)	32.93 (8.9)	*F*(3, 73)=0.46	0.713
Study 2 (Gaza War Exposed)	39.68 (8.6)	45.06 (10.7)	40.80 (9.9)	37.53 (10.7)	*F*(3, 63)=1.66	0.184
(Gaza War Unexposed)	39.62 (9.8)	38.69 (10.1)	38.73 (9.4)	36.60 (9.1)	*F*(3, 70)=0.39	0.764
Sex, women, No. (%)						
Study 1 (Second Lebanon War)	19 (70.4)	12 (66.7)	10 (58.8)	12 (80.0)	χ^2^ (3, *N*=77)=1.74	0.629
Study 2 (Gaza War Exposed)	17 (89.5)	10 (62.5)	9 (60.0)	14 (82.4)	χ^2^ (3, *N*=67)=5.29	0.128
(Gaza War Unexposed)	20 (76.9)	8 (61.5)	8 (53.3)	15 (75.8)	χ^2^ (3, *N*=74)=3.15	0.368
Marital status, married, No. (%)						
Study 1 (Second Lebanon War)	19 (70.4)	12 (66.7)	9 (52.9)	10 (66.7)	χ^2^ (3, *N*=77)=1.47	0.690
Study 2 (Gaza War Exposed)	15 (78.9)	8 (50.0)	11 (73.3)	13 (76.5)	χ^2^ (3, *N*=67)=4.20	0.241
(Gaza War Unexposed)	14 (53.8)	7 (53.8)	12 (80.0)	13 (65.0)	χ^2^ (3, *N*=74)=3.24	0.355
Religiosity, secular, No. (%)						
Study 1 (Second Lebanon War)	25 (92.6)	14 (77.8)	13 (76.5)	14 (100.0)	χ^2^(3, *N=*76)=5.80	0.122
Study 2 (Gaza War Exposed)	15 (78.9)	13 (81.3)	11 (73.3)	12 (70.6)	χ^2^(3, *N=*67)=0.66	0.881
(Gaza War Unexposed)	16 (61.5)	12 (92.3)	14 (93.3)	16 (80.0)	χ^2^(3, *N*=74)=7.85	0.049
Profession, physicians, No. (%)						
Study 1 (Second Lebanon War)	12 (44.4)	9 (50.0)	8 (47.1)	7 (46.7)	χ^2^(3, *N*=77)=0.14	0.987
Study 2 (Gaza War Exposed)	5 (26.3)	6 (37.5)	5 (33.3)	5 (29.4)	χ^2^(3, *N*=67)=0.56	0.905
(Gaza War Unexposed)	7 (26.9)	8 (61.5)	9 (60.0)	9 (45.0)	χ^2^(3, *N=*74)=6.24	0.101

*Note*: Marital status was divided into married and unmarried. Religiosity was divided into secular and religious.

There were no significant differences on age, sex, marital status, profession, and religiosity between the affective types. One exception was a significant difference in the distribution of religiosity among the affective types in Study 2 unexposed personnel; χ^2^ (3, *N*=74)=7.85, *p*<0.05. A lower percentage of seculars were found in the negative congruent type compared to the other types (for more details, see Table 1).

Table 2 presents the descriptive statistics and ANCOVA results when comparing the affective types in the general level of posttraumatic symptoms.

**Table 2:** Univariate analysis of covariance comparing the affective types on general level of posttraumatic symptoms

Source	Negative congruent	Positive congruent	Deflated incongruent	Inflated incongruent	Difference test	Partial η^2^
Study 1 (Second Lebanon War) Mean (SD)	30.15 (13.9)	11.89 (12.1)	12.24 (9.5)	30.64 (13.1)	*F*(3, 72)=14.32***	0.39
Study 2 (Gaza War Exposed) Mean (SD)	28.05 (13.1)	17.25 (8.7)	16.33 (9.8)	28.88 (14.4)	*F*(3, 58)=3.66*	0.16
Study 2 (Gaza War Unexposed) Mean (SD)	24.96 (14.2)	7.00 (7.2)	5.27 (7.5)	12.65 (9.2)	*F*(3, 65)=12.85***	0.37

*Note*: Age, gender, marital status, religion, and profession were served as covariates in this analysis. Marital status was divided into married and unmarried, religion was divided into secular and religious.

* *p*<0.05.

** *p*<0.01.

*** *p*<0.001.

After controlling for demographics, the affective types significantly differed in the general posttraumatic symptoms score: *F* (3, 72)=14.32, *p*<0.001, $\eta_{\text{p}}^{2}$=0.39 for Study 1; *F* (3, 58)=3.66, *p*<0.05, $\eta_{\text{p}}^{2}$ =0.16 for Study 2 exposed personnel; and *F* (3, 65)=12.85, *p*<0.001, $\eta_{\text{p}}^{2}$=0.37, for Study 2 unexposed personnel.

Table 3 presents the descriptive statistics and the ANCOVA results for the analyses that compared the affective types in specific posttraumatic symptoms (intrusion, avoidance, and hyperarousal).

**Table 3:** Multivariate analysis of covariance comparing the affective types on specific posttraumatic symptoms

Source	Negative congruent	Positive congruent	Deflated incongruent	Inflated incongruent	Difference test	Partial η^2^
Study 1 (Second Lebanon War)						
Mean (SD)						
Intrusion	1.42 (1.0)	0.51 (0.6)	0.48 (0.5)	1.37 (0.9)	*F*(3, 66)=8.58***	0.28
Avoidance	1.07 (0.6)	0.56 (0.7)	0.59 (0.7)	1.37 (0.7)	*F*(3, 66)=5.51**	0.20
Hyperarousal	1.72 (0.9)	0.57 (0.6)	0.62 (0.6)	1.46 (0.8)	*F*(3, 66)=13.17***	0.38
Study 2 (Gaza War Exposed)						
Mean (SD)						
Intrusion	1.36 (0.8)	0.56 (0.5)	0.68 (0.6)	1.18 (0.8)	*F*(3, 57)=3.73*	0.16
Avoidance	0.93 (0.6)	1.09 (0.5)	0.88 (0.6)	1.27 (0.8)	*F*(3, 57)=1.32	0.07
Hyperarousal	1.61 (0.8)	0.67 (0.4)	0.66 (0.4)	1.56 (0.6)	*F*(3, 57)=11.06***	0.37
Study 2 (Gaza War Unexposed)						
Mean (SD)						
Intrusion	1.21 (0.8)	0.30 (0.3)	0.22 (0.4)	0.53 (0.5)	*F*(3, 64)=9.38***	0.31
Avoidance	1.14 (0.8)	0.45 (0.6)	0.36 (0.6)	0.71 (0.6)	*F*(3, 64)=6.39**	0.23
Hyperarousal	1.01 (0.8)	0.17 (0.1)	0.11 (0.2)	0.46 (0.4)	*F*(3, 64)=10.51***	0.33

*Note*: Age, gender, marital status, religion, and profession were served as covariates in this analysis. Marital status was divided into married and unmarried, religion was divided into secular and religious.

* *p*<0.05.

** *p*<0.01.

*** *p*<0.001.

The affective types significantly differed on all three posttraumatic subscales with one exception: the affective types did not significantly differ in avoidance scores in Study 2 exposed personnel. A series of contrast analyses showed that in all the cases where a significant effect was observed, the negative congruent type reported more posttraumatic symptoms than the positive congruent and deflated incongruent types. In addition, the negative congruent type reported significantly more posttraumatic symptoms than the inflated incongruent type, but only among Study 2 unexposed personnel.

Table 4 presents the distribution of participants with clinical level of posttraumatic symptoms among the affective types.

**Table 4:** The distribution of participants with clinical level of posttraumatic symptoms among the affective types

	Negative congruent	Positive congruent	Deflated incongruent	Inflated incongruent
	
	*n* (%)	*n* (%)	*n* (%)	*n* (%)
IES-R<33 *n* (%)				
Study 1 (Second Lebanon War)	16 (59.3)	17 (88.9)	17 (100.0)	9 (60.0)
Study 2 (Gaza War Exposed)	11 (57.9)	15 (93.8)	13 (86.7)	11 (64.7)
(Gaza War Unexposed)	18 (69.3)	13 (100.0)	15 (100.0)	20 (100.0)
IES-R≥33 *n* (%)				
Study 1 (Second Lebanon War)	11 (40.7)	2 (11.1)	0 (0.0)	6 (40.0)
Study 2 (Gaza War Exposed)	8 (42.1)	1 (6.2)	2 (13.3)	6 (35.3)
(Gaza War Unexposed)	8 (30.7)	0 (0.0)	0 (0.0)	0 (0.0)

*Note*: IES-R=Impact of Event Scale – Revised.

Among exposed personnel in both studies there was a significantly higher proportion of participants with clinical level of posttraumatic symptoms among the negative congruent and the inflated incongruent types compared to the positive congruent and deflated incongruent types: χ^2^ (3, *N*=77)=13.00, *p*<0.01 and χ^2^ (3, *N*=67)=7.93, *p*<0.05 for Study 1 and Study 2 exposed personnel, respectively. Among Study 2 unexposed personnel, a significant higher proportion of participants were with clinical level of posttraumatic symptoms among the negative congruent type compared to the three other types: χ^2^ (3, *N*=74)=16.56, *p*=0.001. Actually, among unexposed personnel, none of the participants in the other types were above the clinical cutoff score.

## Discussion

Two studies, one during the Second Lebanon War and one during Operation Cast Lead, examined differences in posttraumatic symptoms among hospital personnel divided into different affective types.

In accordance with the research hypothesis, among exposed personnel, it was found that a significantly lower proportion of positive congruent participants had a clinical level of posttraumatic symptoms compared to negative congruent participants (for similar results see Zeidner, 2006). Although not hypothesized, a significantly lower proportion of positive congruent exposed personnel had a clinical level of posttraumatic symptom level compared to inflated incongruent exposed personnel. It was found that a significantly lower proportion of deflated incongruent exposed personnel had a clinical level of posttraumatic symptoms compared to negative congruent and inflated incongruent exposed personnel. Similar results were found when examining the three PTSD symptom clusters. While it is easy to understand why PA is associated with higher resilience (lower level of PTSD symptoms) in front of adversity (Tugade & Ferdrickson, 2004; Zeidner, 2006), it is less clear how affective deflation is associated with lower level of PTSD symptoms. All the more so, the literature tends to relate affective numbness with dissociative phenomena and in turn to higher PTSD levels (Epstein, Fullerton, & Ursano, 1998). In that vein, the present finding seems to be counterintuitive. On the contrary, the literature also suggests that affect constriction can protect against stress. Some authors claim that in traumatic situations some individuals create a protective shield by constricting affect in order to prevent the effect of trauma on their mental state. This is a sort of adaptive dissociation that protects the self from traumatic exposure (Lifton, 1988). Accordingly, it was found that the deflated incongruence affect type was related to a higher level of functioning and to a decreased risk of having pain or somatization disorder compared to the negative congruent affect type among fibromyalgia patients (Hassett et al., 2008). It is possible that due to its protective mode of adaptation this type has advantages during prolonged stress situations.

Although previous studies found the inflated incongruent type to be adaptive in stress, as it may signal high mental complexity (Folkman, 1997; Larsen et al., 2003), it is possible that under acute and prolonged stress, affective inflation reflects emotional confusion and it has some advantages only under intermediate stress such as seen in the unexposed group. Larsen and his colleagues (2001) speculated that emotional inflation is most adaptive during high stress situations, but they also mentioned it as an inconvenient experience. Such an intensive, prolonged, and dialectical emotional reaction may hamper functioning among exposed hospital personnel who need to remain focused on their activities and operate as calmly as possible. Accordingly, it was found that fibromyalgia patients with an inflated affect type reported poorer functioning compared to patients with a positive congruent affect type (Hassett et al., 2008). Still, as unexposed personnel with an inflated incongruent type reported less posttraumatic distress than their negative congruent type counterparts, it is possible that inflated emotional reaction may be less detrimental for hospital personnel operating in routine conditions.

The present findings enhance the understanding that a positive affective types, represented by the positive congruent exposed participant, as well as affective numbness, possibly represented by the deflated incongruent exposed participant, are two different successful modes of adaptation for hospital personnel working in conditions of extreme and prolonged stressful situations. Naturally, our findings need to be further replicated before any firm recommendations could be made regarding the specific interventions aimed to help medical crews in times of stress. However, our findings do suggest that high NA has a detrimental effect for exposed hospital personnel regardless of their PA level (as both negative congruent and inflated incongruent participants reported more distress than their counterparts).

This research has certain limitations. First, the study groups were small and represented a random, but not a representative, sample of hospital personnel. Second, the CES-D is a less prevalent index of PA and contained only three items in our study due to low internal reliability.

Still, this study also has several strengths. It is unique in its population, as medical personnel during prolonged war situations are understudied. The differential examination of affect in the context of ongoing war stress is also understudied. Future studies need to examine the modes of adaptation of these affective types, for example, whether positive congruency mainly reflects positive restructuring, optimism, denial, cynicism, or other mechanisms. Such findings might help promote beneficial interventions for each affective type.

To sum up, it seems that during extreme and prolonged war stress, a positive outlook (see Fredrickson et al., 2003) as well as a detached and a less emotional approach (Hassett et al., 2008) were found to be related to a lower level of PTSD symptoms. Although previous findings reported that the first affective reaction was related to resiliency after the end of the stressful event (Zeidner, 2006), the results are less consistent regarding the advantage of the latter reaction after the stressful event ceased (Epstein et al., 1998). This differentiation needs to be investigated further in the future.
